# Role of airway lactoperoxidase in scavenging of hydrogen peroxide damage in asthma

**DOI:** 10.4103/1817-1737.33698

**Published:** 2007

**Authors:** Amina Hamed Ahmad Al Obaidi

**Affiliations:** *Department of Biochemistry, College of Medicine, Tikrit University, Iraq*

**Keywords:** Asthma, lactoperoxidase, pathogenesis

## Abstract

Hydrogen peroxide (H_2_O_2_) that is mainly generated by neutrophils and eosinophils in asthma is known to be damaging to the airway and to contribute to airway inflammation. The purpose of the present study was to determine the contribution and the role of lactoperoxidase in scavenging airway hydrogen peroxide, in order to propose a therapeutic approach for asthma. The study was an open clinical trial. Twenty-five nonsmoking asthmatic patients were included in the study. Of them, 16 patients (64%) were male and 9 (36%) were female, with age ranging from 29 to 48 years (45.13 ± 4.6). Of the 25 patients included in the study, only 16 patients completed the study; and they were eligible for analyses. Exhaled breath condensate was collected from all patients at the time of entering the study; and 2, 4 and 8 weeks later. All patients received dapson as a lactoperoxidase inhibitor at a dose of 50 mg daily for 8 weeks. The study was conducted during the period from January 2006 to end of October 2006. H_2_O_2_ concentration was determined by an enzymatic assay. Determination of exhaled breath condensate for hydrogen peroxide concentration after 8 weeks of dapson usage indicated an increase (1.05 ± 0.36 μM; 95% CI, 0.89-1.21) as compared to that at baseline (*P* < 0.0001), 2 weeks (*P* < 0.001) and 4 weeks (*P* > 0.05). The increase in hydrogen peroxide concentration in exhaled breath condensate after inhibition of lactoperoxidase by dapson advocates a potential role for lactoperoxidase in scavenging of hydrogen peroxide in asthmatic airway.

Eosinophil peroxidase has been implicated in promoting oxidative tissue damage in a variety of inflammatory conditions, including asthma.[1] Hydrogen peroxide was detected at sixfold higher levels in breath condensate from patients with asthma compared to normal subjects.[2]

Several studies have investigated the role of enzymes in scavenging reactive oxygen species and hydrogen peroxide in particular, and they have suggested that glutathione peroxidase is an important scavenger in the airway.[3,4] However, others found that airway lactoperoxidase was the most important hydrogen peroxide scavenger in sheep airway mucus when only tracheal secretions were collected and examined.[5,6]

The protection of airway epithelium from damage that was induced by hydrogen peroxide needs a balance between enzymatic and nonenzymatic antioxidant defense molecules in the airway.[7] The results of a recently reported study suggested that lactoperoxidase was the major scavenger of hydrogen peroxide in normal human airway secretions.[8] The regulation of airway hydrogen peroxide is important during allergic responses because aerosolized catalase can block allergen-induced hyper-responsiveness in sheep.[9]

Dapson is a pharmacological medication most commonly used for the treatment of Mycobacterium leprae infection as antibacterial dapson inhibits bacterial synthesis of dihydrofolic acid. When used for treatment of skin conditions in which bacteria do not have a role, the mechanism or action of dapson is less well understood. Being used in leprosy, dapson can also be used to treat dermatitis herpetiformis and other skin conditions, including lichen planus.[10] Dapson oral therapy in a dose of 100 mg twice daily for 6-13 months suggests steroid-sparing effects in chronic asthma.[11–13] The wide range of side effects of this drug limited its use as a therapeutic approach for the above listed diseases.[12,13]

As reported, it appears that hydrogen peroxide produced in the airway was mainly consumed by lactoperoxidase.[5,6] The hypothesis was that does lactoperoxidase system may play a role in the pathogenesis of asthma. Thus the purpose of this study was to examine the contribution of lactoperoxidase in exhaled breath condensate following administration of lactoperoxidase inhibitor.

## Materials and Methods

### Patients

Twenty-five nonsmoking asthmatic patients were included in the study. Asthma diagnosis was established according to the National Heart, Lung and Blood Institute guidelines for the diagnosis and management of asthma.[14] The patients were recruited from outpatient's clinic of Asthma and Allergy Centre. The study was an open clinical study. The patients were selected by defined criteria. Then, the potential participants were invited to participate in the study. The participants were then included in the study randomly. The clinical supervision, follow-up and rescue in case of exacerbation was the responsibility of a consultant physician. Complete hematological and liver function tests were performed for all patients before enrollment in the study and every 2 weeks, in order to follow up and determine the side effects of the drug. Exhaled breath condensate was collected from all patients at the time of entering the study; and 2, 4 and 8 weeks later to determine H_2_O_2_ concentration. All patients received dapson (Glaxo Smith) as lactoperoxidase inhibitor at a dose of 50 mg daily for 8 weeks. The study was conducted during the period from January 2006 to end of October 2006. The study was approved by the ethics committee of our college, and written consent was obtained from all participating subjects.

### Lung function test

Lung function (FEV1 predicted percent) was measured by dry spirometry (Autospheror, Discom-14, Chest Corporation, Japan). The values obtained were used for diagnosis and follow-up of patients during the study period.

### Hydrogen peroxide measurement

Expired breath condensate was collected by using a glass condensing device that was placed in a large chamber with ice. After rinsing their mouth, subjects breathed tiredly with normal frequency through a mouthpiece for 20 min while wearing a nose clip. H_2_O_2_ assay was carried out by using colorimetric assay, as described previously.[15] Briefly, 100 μl of condensate was mixed with 100 μl of tetramethylbenzidine in 0.42 mol/L citrate buffer, pH 3.8, and 10 μl of horseradish peroxidase (52.5 U/ml). The samples were incubated at room temperature for 20 min, and reaction was stopped by addition of 10 μl 18N sulfuric acid. The reaction product was measured spectrophotometrically (Spectrophotometer, LKB, Biochrom, Cambridge, England) at 450 nm. A standard curve of H_2_O_2_ was performed for each assay.

### Statistical analysis

Data concerning the comparisons among the various parameters in the study groups are given as mean (SD) with 95% confidence intervals for the differences. Wilcoxon rank sum test was used for comparison of the groups for significant testing.

## Results

**Figure 1:**
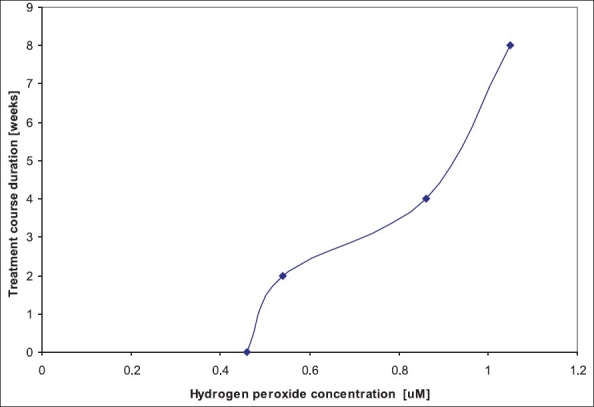
Hydrogen peroxide concentration in expired breath condensate following dapson treatment in asthmatic patients

Twenty-five patients were included in the study; of them, 16 patients (64%) were male and 9 patients (36%) were female. In addition, only 16 patients completed the designed course and were eligible for analysis. Patients' age ranged between 29 and 48 years (45.13 ± 4.6), and all of them were moderate persistent asthmatic patients. At the baseline, the mean expired breath condensate hydrogen peroxide was 0.46 ± 0.16 μM (95% CI, 0.37-0.54). After 2 weeks of treatment with dapson, the mean hydrogen peroxide in exhaled breath condensate increased to 0.54 ± 0.23 μM (95% CI, 0.44-0.64, *P* > 0.05). The increase in exhaled breath condensate hydrogen concentration was highly significant (0.86 ± 0.28 μM; 95% CI, 0.74-0.98; *P* < 0.001) after 4 weeks of treatment with dapson. Determination of exhaled breath condensate concentration after 8 weeks of dapson usage indicated an increase in H_2_O_2_ (1.05 ± 0.36 μM; 95% CI, 0.89-1.21) as compared to that at baseline (*P* < 0.0001), 2 weeks (*P* < 0.001) and 4 weeks (*P* > 0.05). Thus dapson usage results in an increase in exhaled breath condensate H_2_O_2_ concentration, indicating that there is a potential role for lactoperoxidase in scavenging of hydrogen peroxide [Table 1 and Figure 1].

**Table 1:** Exhaled breath condensate hydrogen peroxide concentration [μM] in patients treated with dapson

Period	Mean hydrogen peroxide concentration [16 patients]	SD	95% CI
Baseline	0.46	0.16	0.37 - 0.54
2 weeks	0.54	0.23	0.44 - 0.64
*P* value [2 weeks Vs baseline]	> 0.05		
4 weeks	0.86	0.28	0.74 - 0.98
*P* value [4 week Vs baseline]	<0.001		
8 weeks	1.05	0.36	0.89 - 1.21
*P* value [8 weeks Vs baseline]	<0.0001		

## Discussion

Hydrogen peroxide is known to play an important role in airway homeostasis.[16] For this reason its levels, and thus its synthesis and consumption, are important mechanisms for controlling airway functions.[17] Lactoperoxidase as a major macromolecular consumer of hydrogen peroxide in airway secretions was identified in sheep airway secretions.[16]

In this study, dapson usage in asthmatic patients led to an increase in expired breath condensate hydrogen peroxide, which correlated with duration of dapson treatment. This indicates that the increase of expired breath condensate hydrogen peroxide was due to inhibition of lactoperoxidase activity induced by dapson treatment. The above effect was neither due to depression of mucocilliary velocity nor due to inhibition of myeloperoxidase.[18] The dapson effect was reversed by adding exogenous lactoperoxidase.[19] Normal human tracheal secretions scavenge hydrogen peroxide both in nonenzymatic (approximately 20%) and enzymatic (approximately 80%) fashion, and the major enzymatic consumption was due to lactoperoxidase.[8]

The balance of enzymatic and nonenzymatic processes to scavenge hydrogen peroxide in normal human airways was examined by EL-Chemaly *et al.*[8] Their results indicated that lactoperoxidase was the major enzymatic hydrogen consumer in normal airway secretions. In asthmatic patients, oxidative stress plays an important pathogenetic role.[20,21] The activation of inflammatory cells such as eosinophils is seen in patients with asthma. Activated inflammatory cells respond with a respiratory burst, which results in the production of reactive oxygen species such as hydrogen peroxide.[22] Thus with dapson treatment, which inhibited lactoperoxidase, hydrogen peroxide in expired breath condensate increased in a steady state pattern with the course duration. This indicates the role of lactoperoxidase in scavenging of hydrogen peroxide and in asthma pathogenesis.

The lactoperoxidase antibacterial system increases activity with decreasing pH.[17] The lactoperoxidase system, when supplied with adequate hydrogen peroxide, is effective at pH 6.8, the estimated pH of airway surface liquid *in vivo.*[23,24] Any lowering of the pH of airway secretions would increase lactoperoxidase-mediated antibacterial activity,[17] indicating increased scavenging activity of hydrogen peroxide by lactoperoxidase. Thus any alteration in normal airway concentration of substrate (for example, hydrogen peroxide) and acidity would be expected to change the activity of lactoperoxidase system, with possible harmful effects.

Prior reported studies indicated that there was an increase in expired breath condensate hydrogen peroxide concentration in asthma.[2,20,25–27] Studies have shown that pretreating sheep airway with a hydrogen peroxide scavenger such as catalase can significantly decrease bronchial hyper-responsiveness in response to allergen.[9] In addition, the presence of pathophysiologically relevant levels of peroxides and hydrogen peroxide reversibility ‘nitric oxide’-dependent bronchodilation of preconstricted tracheal ring.[28] Therefore, it is clear that reactive oxygen species, including hydrogen peroxide, play a major role in asthma, and a better understanding of the mechanisms by which the airway scavenges hydrogen peroxide in normal and abnormal conditions is crucial; as it will help to understand asthma pathogenicity and possibly lead to novel therapeutic approaches.

In conclusion, this study indicated that airway lactoperoxidase may play an important role in the scavenging of hydrogen peroxide damage in asthma. The hypothesis that was suggested depending on the findings of this study treats the asthmatic with lactoperoxidase aerosol, controls disease pathogenicity and reduces the damaging effect of increased hydrogen peroxide. This hypothesis needs to be evaluated in an animal model to enable it to make a contribution that may help in asthma treatment.
